# Socio-economic inequalities in non-use of modern contraceptives among young and non-young married women in India

**DOI:** 10.1186/s12889-023-15669-w

**Published:** 2023-05-01

**Authors:** Shobhit Srivastava, Parimala Mohanty, T. Muhammad, Manish Kumar

**Affiliations:** 1 International Institute for Population Sciences, Mumbai, Maharashtra 400088 India; 2 Institute of Medical Sciences & Sum Hospital, Siksha “O” Anusandhan Deemed to Be University, Bhubaneswar, Odisha India

**Keywords:** Modern contraceptives, Socioeconomic inequality, Young and non-young women, India

## Abstract

**Background:**

It is documented that married women do not utilize contraceptive methods, because of the fear of adverse effects, no or seldom sexual interaction; perception that they should not use contraception during breastfeeding, postpartum amenorrhea, or dissatisfaction with a specific method of contraception. The current study aimed to examine the socio-economic inequalities associated with the non-use of modern contraceptive methods among young (15-24 years) and non-young (25-49 years) married women and the contributing factors in those inequalities.

**Methods:**

The present study utilized the cross-sectional data from the fourth round of the National Family Health Survey (NFHS-4) with a sample of 499,627 women who were currently married. The modern methods of family planning include sterilization, injectables, intrauterine devices (IUDs/PPIUDs), contraceptive pills, implants, the standard days method, condoms, diaphragm, foam/jelly, the lactational amenorrhea method, and emergency contraception. Multivariable logistic regression analysis was used to estimate the odds of non-use of modern contraceptive methods according to different age groups after controlling for various confounding factors. Additionally, concentration curve and Wagstaff decomposition method were used in the study.

**Results:**

The prevalence of non-use of modern contraceptive use was higher among women from young category (79.0%) than non-young category (45.8%). The difference in prevalence was significant (33.2%; *p* < 0.001). Women from non-young age group had 39% significantly lower odds of non-use of modern contraceptive use than women from young age group (15–24 years) [AOR: 0.23; CI: 0.23, 0.23]. The value of concentration quintile was -0.022 for young and -0.058 for non-young age groups which also confirms that the non-use of modern contraceptives was more concentrated among women from poor socio-economic group and the inequality is higher among non-young women compared to young women. About 87.8 and 55.5% of the socio-economic inequality was explained by wealth quintile for modern contraceptive use in young and non-young women. A higher percent contribution of educational status (56.8%) in socio-economic inequality in non-use of modern contraceptive use was observed in non-young women compared to only -6.4% in young women. Further, the exposure to mass media was a major contributor to socio-economic inequality in young (35.8%) and non-young (43.2%) women.

**Conclusion:**

Adverse socioeconomic and cultural factors like low levels of education, no exposure to mass media, lack of or limited knowledge about family planning, poor household wealth status, religion, and ethnicity remain impediments to the use of modern contraceptives. Thus, the current findings provide evidence to promote and enhance the use of modern contraceptives by reducing socioeconomic inequality.

## Background

The use of contraceptives is intricately linked to permitting people for making potential choices regarding their reproductive life and childbirth preference [1]. Modern contraceptive has long been recognized as one of the pivotal cost-effective strategies for boosting socio-economic growth through education, gender equality, human rights, and reduction of sexually transmitted diseases and poverty [2, 3]. Despite the rising popularity of contraceptive and the desire for family planning, in the year 2019 globally, only an estimated 8 crores of young and non-young women from 15 to 49 years used modern contraceptives leaving 27 crores with an unmet need [4]. In the low- and middle-income countries more than 20 crores of women wanting to prevent pregnancy do not use contraceptives contributing to 84 percent of unintended pregnancies [5]. Unmet family planning needs are highest among women under the age of 20 and lowest among women 35 and older throughout the world [6].

India has created conducive policies implementations for the use of contraceptive [7]. Back in the year 1952, India was the first country to implement a family planning program, and priotised family planning as an integral part of many national plans and reproductive and child health programs [8]. To increase the use of family planning services in the country, many initiatives have been used over time, including a coercive target strategy, contraceptive-specific incentives, and a family planning camp approach [9]. It has been found, that the unmet need for family planning has decreased over the past 25 years, especially following the International Conference on Population and Development in Cairo (ICPD-1994), from 20.3% in 1992–1993 to 12.9% in 2015–2016 [10]. The need for family planning met by modern methods increased from 58.6 to 71.8% during the period of 1990–2015, while the unmet need for modern methods declined from 25.4% in 1990 to 20.4% in 2015 [11].

Various determinants are likely to influence contraceptive use, ranging at different levels from, individual-related factors, household-related factors, community-related factors, system-related factors, or the interplay of combinations of these factors [12]. Individual factors include education level, partner violence, fertility preferences, and media exposure [12, 13]; household factors include, spousal communications on family planning, and autonomy [14, 15]; community-related factors include caste, religion, place of residence and cultural norms pertaining to family planning [16, 17]. There are cross-country as well as within-country disparities, with lower levels of contraceptive use among poorer, illiterate, rural, and younger women [18]. Further these disparities are most pronounced in southern region of Asia, including India [19]. Studies show that in the Indian society many factors like urban vs rural residence, socioeconomic factors like household wealth and media exposure are likely to influence contraceptive use [11, 18, 20]. Multiple pieces of research in India have extensively focused on the trend of contraceptive use, differentials, and its predictors [11, 21]. However, the level of economic inequality in the use of modern contraceptives and its relationship remain unknown [22]. To understand health disparities, it is suggested to include aggregate measures of socioeconomic status [23].

Evidence suggests that youth faces high sexual and reproductive health risks and their age group is an important social determinant of health [7, 24]. A study comparing contraceptive use in adolescent girls (ages 15–19 years) and adult women (ages 20–34) in 103 low- and middle-income countries between 2000 and 2017 found that adolescent girls continue to fall behind adult women in contraceptive use [25]. Another study between 1992–93 and 2015–16, found the usage of modern contraception among married adolescents grew from 4 to 10%, however being uneducated, residing in rural areas, backward classes, poorest wealth quintile, women with no child, and ones with no mass media exposure were shown to have low uptake of modern contraceptives [26]. Throughout the literature, inequality of these economic and socio-cultural factors had an influence on the use of modern contraceptives.

**Fig. 1:**
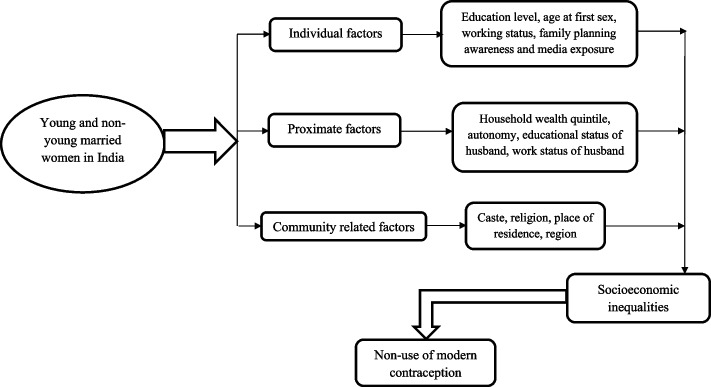
Conceptual framework of the study

We found relatively scarce work as most of the previous studies from India only looked at the overall family planning services, levels and trends in contraceptive prevalence and predictors of contraceptives use [7, 11, 20, 26]. Therefore, to our knowledge, ours is one among the few studies from India to report various factors that determine the non-use of modern contraceptives and their associated inequalities among young and non-young women. Generating more clear evidence will have significant policy consequences for achieving SDG 3.7, which targets universal access to family planning services and promote healthy lives and well-being [27]. Thus, this study aimed to examine the factors contributing to the socio-economic inequalities associated with non-use of modern contraceptive methods among young and non-young married women in India. Based on the above literature, a conceptual framework has been developed and summarised in Fig. 1. Our study's conclusions may also have significant policy implications for those stakeholders and decision-makers working to improve and promote modern contraceptives by reducing the related socio-economic inequality among young non-young women in India.

The study hypothesizes that.

- H1: there is significant wealth-based inequality for the non-use of modern contraceptives among young and non-young married women in India.
- H2: there is a higher concentration of non-use of modern contraceptives among youth than non-youth from higher socioeconomic status.
- H3: low levels of wealth, low education working status, exposure to mass media, wealth, social class, and place of residence are positively associated with non-use of modern contraceptives among young and non-young married women in India.

## Materials and methods

### Data

The present study utilized the cross-sectional data from the fourth round of the National Family Health Survey (NFHS-4) conducted during 2015–16. The NFHS-4 is a large-scale cross-sectional, and nationally representative sample survey carried out under the stewardship of the Ministry of Health and Family Welfare (MoHFW), Government of India. NFHS-4 provides self-reported information about demographic, socio-economic, maternal, and child health outcomes, family planning, and reproductive health. In NFHS-4, a multistage stratified random sampling method was adopted for the collection of data. It adopted three-stage sampling in urban area and two-stage sampling design in the rural area. In urban areas, in first stage, wards were selected with Probability proportional to size (PPS) sampling. In the next stage, one census enumeration block (CEB) was selected randomly from each sampled ward. In the final stage, household were selected from each selected CEB. In rural areas, villages referred as Primary Sampling Units (PSUs) were selected in the first stage, followed by the selection of the households in the selected villages using systematic random sampling. Details of the sample size, design, and sample weights in NFHS-4 were published elsewhere [10]. NFHS-4 surveyed a total of 699,686 women aged 15–49 in 601,509 households, with a response rate of 97 percent.

### Final sample size

The effective sample size for the present study was 499,627 women who were currently married. Moreover, the number of women who were currently married and aged 15–24 years (young) was 94,034 and the number of women who were currently married and aged 25–49 years (non-young) were 405,593.

### Measures

#### Dependent variable

The dependent variable in this study was "modern contraceptive use". Two questions were used to determine the women utilizing modern contraceptive methods: (1) Are you currently doing something or using any method to delay or avoid getting pregnant? If yes: (2) Which method, are you using? The modern methods of family planning include sterilization, injectables, intrauterine devices (IUDs/PPIUDs), contraceptive pills, implants, the standard days method, condoms, diaphragm, foam/jelly, the lactational amenorrhea method, and emergency contraception. Women utilizing the modern contraceptive methods were coded as '1', otherwise '0'.

### Explanatory variables

Various explanatory characteristics related to women, husbands, and households were included in the analysis. Women's characteristics include age at first sex (*no sex,* < *18 years,* ≥ *18 years*), educational status (*not educated, primary, secondary, higher*), working status (*currently working, currently not working*), exposure to mass media (*no, yes*), heard family planning on radio last few months (*no, yes*), heard family planning on television last few months (*no, yes*), heard family planning in newspaper/magazine last few months (*no, yes*). Husband's characteristics include educational status (*not educated, secondary, primary, higher*), working status (*currently not working, currently working*). Household characteristics consist of wealth index (*poorest, poorer, middle, richer, richest*), religion (*Hindu, Muslim, others*), caste (*Scheduled Caste, Scheduled Tribe, Other Backward Class, others*), place of residence (*urban, rural*), regions (*north, central, east, northeast, west, south*).

The variable of wealth index was created using the information given in the survey. Households were given scores based on the number and kinds of consumer goods they own, ranging from a television to a bicycle or car, and housing characteristics such as source of drinking water, toilet facilities, and flooring materials. These scores are derived using principal component analysis. National wealth quintiles are compiled by assigning the household score to each usual (de jure) household member, ranking each person in the household population by their score, and then dividing the distribution into five equal categories, each with 20% of the population.

## Statistical analysis

Descriptive analysis was utilized to report the general characteristics of the sample. Proportion tests were utilized to assess the significant difference in the prevalence of non-use modern contraceptive methods among women in young (15–24 years) and non-young age (25–49 years) groups according to different characteristics. Since our dependent variable, non-use of modern contraceptive methods, is binary, logistic regression analysis was used to estimate the odds of non-use of modern contraceptive methods according to different age groups after controlling for various confounding factors.

The concentration index quantifies the degree of socio-economic inequality in the given outcome variable [28]. Due to the binary nature of the dependent variable, we used the corrected concentration index (CCI) that is a rescaled concentration index which ensures the variability of the index within the range of -1 and 1 [29]. The CCI of the variable is given by:

1 $$CCI=\frac{1}{n}{\sum }_{i=1}^{n}\left[\frac{(a-b)}{\left(a-\mu \right)(\mu -b)} (2{r}_{i}-1)\right]$$

where n is the sample size, $\mu$ is the mean non-use of the modern contraception, $a$ and $b$ are the maximum and minimum levels of non-use of modern contraception (i.e., 0 and 1), and ${r}_{i}=i-0.5/n$ is the fractional rank of the individual $i$ in the socio-economic status, with $i=1$ for the poorest and $i=n$ for the richest. The negative (positive) index value implies the pro-poor (pro-rich) inequality in the non-use of modern contraceptive methods. The values are provided for Generalized CCI. As a sensitivity check, we estimated and report CCI using other two approaches of Erreygers normalized CCI and Wagstaff normalized CCI.

### Decomposition of CCI

To determine the contribution of various determinants to socio-economic inequality, CCI was decomposed using the Wagstaff-type decomposition methodology [30]. The Wagstaff-type decomposition technique decomposes Generalized CCI. The equation of the linear relationship of the continuous outcome variable and its k predictors is given as:

2 $${y}_{i}=\alpha +{\sum }_{k}{\beta }_{k}{x}_{ki}+ {\varepsilon }_{i}$$

where ${y}_{i}$ is the outcome variable, ${x}_{k}$ is the set of predictors, and $\varepsilon$ is the error term that follows the normal distribution ${e}_{i}\sim N(0, {\sigma }^{2})$. The overall CCI can be represented as the linear combination of ${CCI}_{k}$ of the determinants and the ratio of the generalized concentration index (GC) of the error term to the mean outcome variable as follows [30]:

3 $$CI=\sum \left(\frac{{\widehat{\beta }}_{k}}{\mu }{\overline{x} }_{k}\right){CCI}_{k}+\frac{{GC}_{\varepsilon }}{\mu }$$

where $\mathrm{CI}$ denotes the overall concentration index, $\upmu$ is the mean of $\mathrm{y}$, ${\overline{\mathrm{x}} }_{\mathrm{k}}$ is the mean of ${\mathrm{x}}_{\mathrm{k}}$, ${\mathrm{C}}_{\mathrm{k}}$ is the normalized concentration index for ${\mathrm{x}}_{\mathrm{k}}$ (defined exactly like CCI), $\frac{{\upbeta }_{\mathrm{k}}{\overline{\mathrm{x}} }_{\mathrm{k}}}{\upmu }$ is the elasticity of outcome variable with the explanatory variables, and ${\mathrm{GC}}_{\upvarepsilon }$ is the generalized CCI for ${\varepsilon }_{i}$ (residual component). Eq. (3) suggests that the concentration index consists of explained and residual (unexplained) components. Since outcome variable is not continuous, we have approximated decomposition analysis by using marginal effects on the logit model. A linear approximation of the non-linear estimation can be represented as:

4 $${\mathrm{y}}_{\mathrm{i}}={\mathrm{\alpha }}^{\mathrm{m}}+{\sum }_{\mathrm{k}}{\upbeta }_{\mathrm{k}}^{\mathrm{m}}{\mathrm{x}}_{\mathrm{ki}}+{\upmu }_{\mathrm{i}}$$

where ${\upbeta }_{\mathrm{k}}^{\mathrm{m}}$ is the marginal effects ($\frac{\mathrm{dy}}{\mathrm{dx}}$) of each x; ${\upmu }_{\mathrm{i}}$ signifies the error term generated by the linear approximation. The concentration index for the outcome variable (y) (in our case, use of modern contraceptive methods) is given as:

5 $$\mathrm{CI}={\sum }_{\mathrm{k}}(\frac{{\upbeta }_{\mathrm{k}}{\overline{\mathrm{x}} }_{\mathrm{k}}}{\upmu }){\mathrm{C}}_{\mathrm{k}}+{\mathrm{GC}}_{\upvarepsilon }/\upmu$$

## Results

Table 1 provides the socio-demographic characteristics of the study participants. A proportion of 40.5 and 40.6% of young and non-young respectively had sex before the age of 18 years. About 17.7 and 36.7% of women were not educated in the young and non-young category, respectively. A proportion 11.2 and 26.2% of women were working in young and non-young category, respectively. Almost 79% of women in both young and non-young category had mass media exposure. Only 16.7 and 18.1% of women from young and non-young categories reported that they heard about family planning on radio. Similarly, a proportion of 56.9 and 58.4% of women reported that they heard about family planning on television. Also, a proportion of 34.2 and 33.9% of young and non-young women heard about family planning through newspapers and magazines.

**Table 1:** Sample characteristics of the study population, 2015–16

Background characteristics	Youth (15–24 years)		Non-youth (25–49 years)	
	**Sample**	**Percentage**	**Sample**	**Percentage**
**Women characteristics**				
**Age at first sex**				
No sex	2,487	2.6	21,909	5.4
< 18 years	38,116	40.5	164,711	40.6
≥ 18 years	53,431	56.8	218,972	54.0
**Educational status**				
Not educated	16,651	17.7	148,803	36.7
Primary	55,213	58.7	156,862	38.7
Secondary	12,353	13.1	59,058	14.6
Higher	9,817	10.4	40,870	10.1
**Working status (last 12 months)**				
Currently working	1,776	11.2	18,579	26.2
Currently not working	14,037	88.8	52,419	73.8
**Exposure to mass media**				
No	19,775	21.0	84,642	20.9
Yes	74,259	79.0	320,951	79.1
**Heard family planning on radio last few months**				
No	78,345	83.3	332,250	81.9
Yes	15,689	16.7	73,343	18.1
**Heard family planning on television last few months**				
No	40,499	43.1	168,918	41.7
Yes	53,535	56.9	236,675	58.4
**Heard family planning in newspaper/magazine last few months**				
No	61,848	65.8	268,006	66.1
Yes	32,186	34.2	137,587	33.9
**Husband characteristics**				
**Educational status**				
Not educated	1,879	11.9	14,294	20.1
Primary	9,468	59.9	35,600	50.1
Secondary	2,041	12.9	10,982	15.5
Higher	2,425	15.3	10,123	14.3
**Working status (last 12 months)**				
Currently not working	861	5.4	2,739	3.9
Currently working	14,952	94.6	68,259	96.1
**Household characteristics**				
**Wealth Index**				
Poorest	19,512	20.8	71,147	17.5
Poorer	22,416	23.8	76,037	18.8
Middle	21,383	22.7	80,825	19.9
Richer	18,532	19.7	86,524	21.3
Richest	12,191	13.0	91,060	22.5
**Religion**				
Hindu	75,807	80.6	331,116	81.6
Muslim	14,468	15.4	51,226	12.6
Others	3,759	4.0	23,252	5.7
**Caste**				
Scheduled Caste	20,760	22.1	80,407	19.8
Scheduled Tribe	10,064	10.7	35,513	8.8
Other Backward Class	40,845	43.4	177,256	43.7
Others	22,365	23.8	112,417	27.7
**Place of residence**				
Urban	24,374	25.9	142,799	35.2
Rural	69,660	74.1	262,794	64.8
**Regions**				
North	11,658	12.4	55,422	13.7
Central	22,007	23.4	90,686	22.4
East	26,389	28.1	88,898	21.9
North East	3,261	3.5	13,673	3.4
West	12,892	13.7	59,152	14.6
South	17,827	19.0	97,762	24.1
**Total**	94,034	100.0	405,593	100.0

Table 2 represents the percentage of women not using modern contraceptives by their background characteristics. It was found that the prevalence of modern contraceptive use was higher among women from young category (79.0%) than non-young category (45.8%). The difference in prevalence was significant (33.2%; *p* < 0.001).

**Table 2:** Percentage of non-use of modern contraceptive methods among currently married women by background characteristics in India, 2015–16

Background characteristics	Youth (15–24 years)	Non-youth (25–49 years)	Differences	*p*-value
	**Percentage**	**Percentage**	**Percentage**	
**Women characteristics**				
**Age at first sex**				
No sex	72.7	47.7	25.0	< 0.001
< 18 years	73.7	40.7	33.0	< 0.001
≥ 18 years	83.1	49.5	33.6	< 0.001
**Educational status**				
Not educated	83.9	47.2	36.8	< 0.001
Primary	77.5	44.3	33.2	< 0.001
Secondary	76.9	40.8	36.1	< 0.001
Higher	81.6	54.1	27.5	< 0.001
**Working status (last 12 months)**				
Currently working	72.4	37.2	35.2	< 0.001
Currently not working	79.0	47.8	31.2	< 0.001
**Exposure to mass media**				
No	86.3	58.4	28.0	< 0.001
Yes	77.0	42.5	34.5	< 0.001
**Heard family planning on radio last few months**				
No	78.7	45.6	33.1	< 0.001
Yes	80.7	47.1	33.6	< 0.001
**Heard family planning on television last few months**				
No	82.4	51.3	31.2	< 0.001
Yes	76.4	41.9	34.5	< 0.001
**Heard family planning in newspaper/magazine last few months**				
No	79.8	46.2	33.6	< 0.001
Yes	77.4	45.0	32.4	< 0.001
**Husband characteristics**				
**Educational status**				
Not educated	81.1	45.6	35.6	< 0.001
Primary	78.2	44.7	33.5	< 0.001
Secondary	73.4	39.6	33.8	< 0.001
Higher	80.4	51.0	29.4	< 0.001
**Working status (last 12 months)**				
Currently not working	81.8	50.0	31.9	< 0.001
Currently working	78.1	44.8	33.3	< 0.001
**Household characteristics**				
**Wealth Index**				
Poorest	84.6	57.7	26.9	< 0.001
Poorer	79.5	46.5	33.0	< 0.001
Middle	77.7	42.1	35.6	< 0.001
Richer	75.9	42.0	33.9	< 0.001
Richest	75.8	42.9	33.0	< 0.001
**Religion**				
Hindu	79.3	44.5	34.8	< 0.001
Muslim	79.2	57.1	22.1	< 0.001
Others	71.9	40.2	31.6	< 0.001
**Caste**				
Scheduled Caste	77.6	43.7	34.0	< 0.001
Scheduled Tribe	81.4	47.2	34.2	< 0.001
Other Backward Class	82.0	46.7	35.3	< 0.001
Others	73.6	45.6	28.1	< 0.001
**Place of residence**				
Urban	75.9	44.0	31.9	< 0.001
Rural	80.1	46.8	33.3	< 0.001
**Regions**				
North	76.3	37.9	38.4	< 0.001
Central	84.9	55.0	29.9	< 0.001
East	77.5	53.8	23.7	< 0.001
North East	71.8	63.4	8.4	< 0.001
West	77.4	36.8	40.6	< 0.001
South	78.2	37.6	40.6	< 0.001
	79.0	45.8	33.2	< 0.001

Difference: %youth—%non-youth; *p*-value based on proportion test

Table 3 reveals logistic regression estimates for non-use of modern contraceptive use among women by their background characteristics. The estimates presented are adjusted estimates. It was found that age was significantly associated with non-use of modern contraceptive among women. That is women from non-young age group (25–49 years) had 39% significantly lower odds of non-use of modern contraceptive than women from young age group (15–24 years) [AOR: 0.23; CI: 0.23, 0.23]. Additionally, education, exposure to mass media, knowledge about family planning, household wealth status, religion and ethnicity were the significant predictors of modern contraceptive use among women.

**Table 3:** Logistic regression estimates for non-use of modern contraceptive methods among currently married women by background characteristics in India, 2015–16

Background characteristics	AOR
	**95% CI**
**Women characteristics**	
**Age group**	
Youth (15–24 years)	Ref
Non-youth (25–49 years)	0.23*(0.23,0.23)
**Age at first sex**	
No sex	Ref
< 18 years	0.63*(0.62,0.65)
≥ 18 years	0.98(0.95,1)
**Educational status**	
Not educated	0.55*(0.54,0.57)
Primary	0.66*(0.64,0.68)
Secondary	0.52*(0.51,0.54)
Higher	Ref
**Exposure to mass media**	
No	1.37*(1.34,1.39)
Yes	Ref
**Heard family planning on radio last few months**	
No	0.83*(0.81,0.84)
Yes	Ref
**Heard family planning on television last few months**	
No	1.37*(1.35,1.39)
Yes	Ref
**Heard family planning in newspaper/magazine last few months**	
No	0.98*(0.96,0.99)
Yes	Ref
**Household characteristics**	
**Wealth Index**	
Poorest	1.37*(1.33,1.41)
Poorer	1.16*(1.14,1.19)
Middle	1.14*(1.11,1.16)
Richer	1.12*(1.1,1.15)
Richest	Ref
**Religion**	
Hindu	Ref
Muslim	1.73*(1.7,1.76)
Others	1.13*(1.1,1.16)
**Caste**	
Scheduled Caste	1.06*(1.03,1.08)
Scheduled Tribe	1.14*(1.11,1.16)
Other Backward Class	1.07*(1.05,1.09)
Others	Ref
**Place of residence**	
Urban	Ref
Rural	0.99(0.97,1)
**Regions**	
North	Ref
Central	1.69*(1.65,1.72)
East	1.74*(1.71,1.78)
North East	2.59*(2.53,2.66)
West	1.06*(1.03,1.08)
South	1.07*(1.05,1.1)

*Ref* Reference, *CI* Confidence Interval; *if *p* < 0.05; *AOR* Adjusted Odds Ratio

**Fig. 2:**
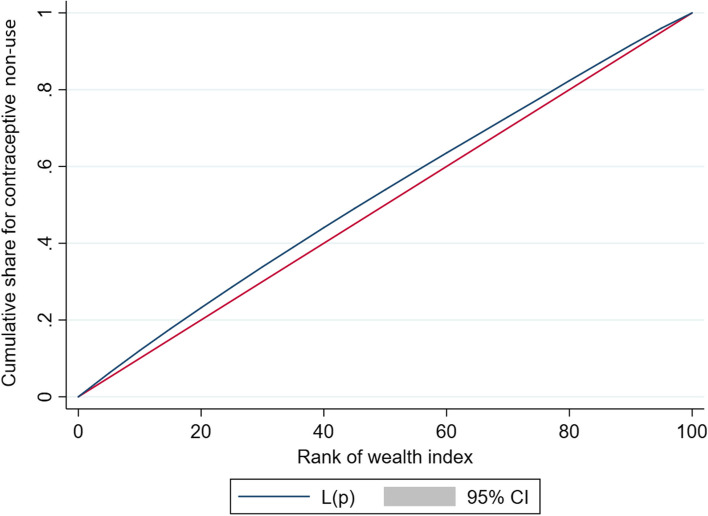
Concentration curve of non-use of modern contraceptive among young married women age 15–24 years

**Fig. 3:**
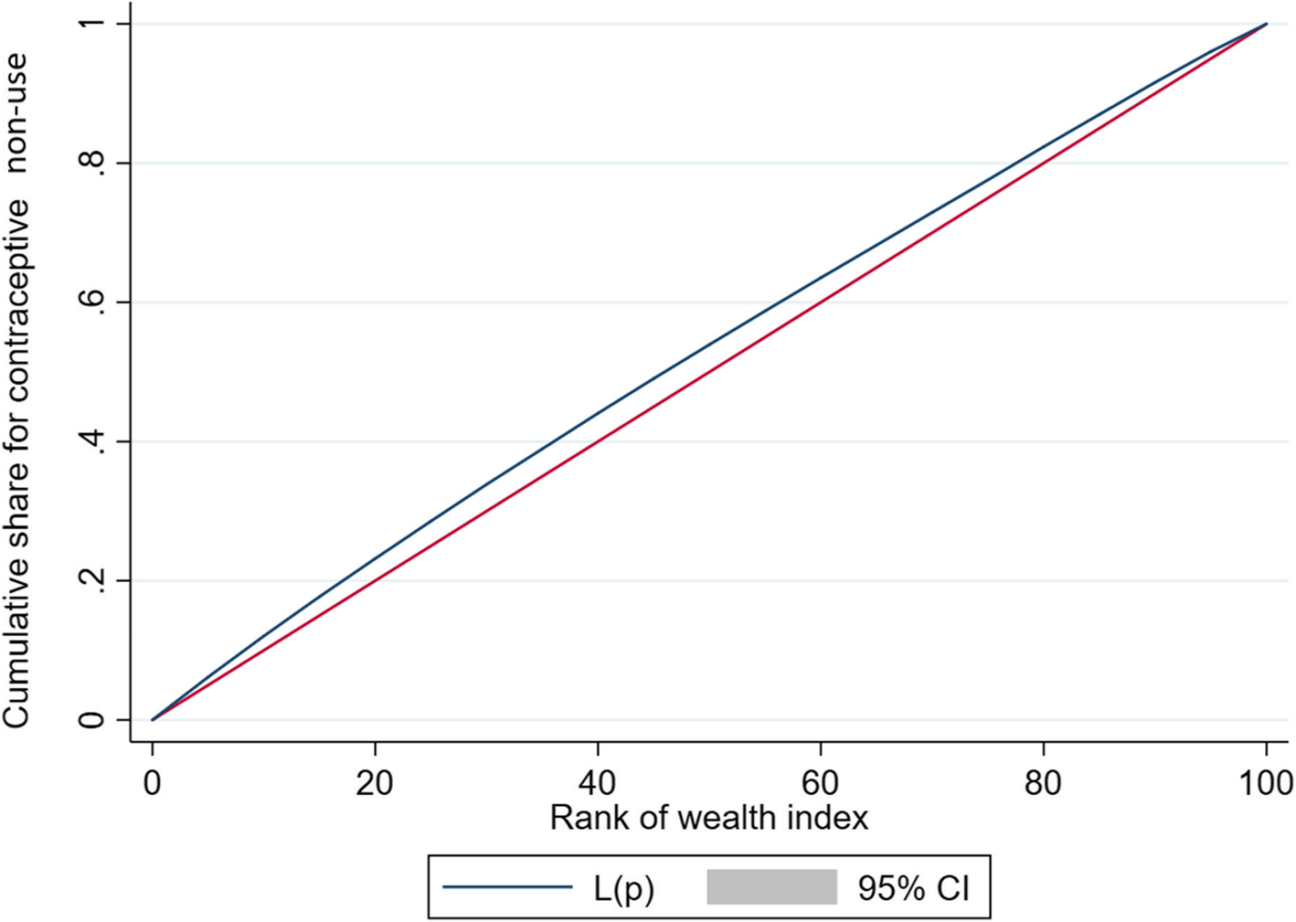
Concentration curve of non-use of modern contraceptive among non-young married women age 25–49 years

Figures 2 and 3 present the concentration curves of non-use of modern contraceptives for young and non-young women, respectively.

Table 4 reveals that non-use of modern contraceptive is concentrated among women from poor socio-economic strata both in young and non-young categories. The value of concentration quintile was -0.022 for young and -0.058 for non-young age groups which also confirms that the non-use of modern contraceptive use was more concentrated among women from poor socio-economic group and the inequality is higher among non-young women compared to young women (difference: 0.036, *p* < 0.001).

**Table 4:** CCI for non-use of modern contraceptive methods among the currently married women in India, 2015–16

Types of CCI	Youth	Non-youth	Difference	*p*-value
Generalized CCI	-0.022	-0.058	0.036	< 0.001
Erreygers normalized CCI	-0.036	-0.053	0.017	< 0.001
Wagstaff normalized CCI	-0.095	-0.067	-0.028	< 0.001

*CCI* Concentration Index

Table 5 represents the decomposition estimates for non-use of modern contraceptive use among young and non-young women. It was found that about 87.8 and 55.5% of the socio-economic inequality was explained by wealth quintile for modern contraceptive use in young and non-young women. A higher percent contribution of educational status (56.8%) in socio-economic inequality in non-use of modern contraceptive use was observed in non-young women compared to only -6.4% in young women. Further, the exposure to mass media was a major contributor to socio-economic inequality in young (35.8%) and non-young (43.2%) women. The knowledge about family planning through television explained 26.9 and 30.8% of the inequality in non-use of modern contraceptive use among young and non-young women, respectively. Additionally, region explained the observed inequality for non-use of modern contraceptive use by about -14.2% in young and 68.4% in non-young women.

**Table 5:** Decomposition estimates for non-usage of modern contraceptive methods among currently married women in India, 2015–16

Background characteristics	Youth (15–24 years)					Non-youth (25–49 years)				
	**Elasticity**	**CCI**	**Absolute CCI**	**% Contribution**		**Elasticity**	**CCI**	**Absolute CCI**	**% Contribution**	
**Women characteristics**										
**Age at first sex**										
No sex										
< 18 years	0.001	-0.168	0.000	1.2		-0.042	-0.160	0.007	-26.4	
≥ 18 years	0.062	0.120	0.007	-43.7	-42.6	-0.002	0.124	0.000	0.8	-25.6
**Educational status**										
Not educated										
Primary	-0.010	0.088	-0.001	5.4		0.018	0.219	0.004	-15.8	
Secondary	-0.005	-0.229	0.001	-7.1		-0.002	-0.125	0.000	-1.2	
Higher	0.002	0.523	0.001	-4.6	-6.4	0.015	0.651	0.010	-39.8	-56.8
**Exposure to mass media**										
No										
Yes	-0.043	0.144	-0.006	35.8	35.8	-0.071	0.154	-0.011	43.2	43.2
**Heard family planning on radio last few months**										
No										
Yes	0.006	0.103	0.001	-3.5	-3.5	0.007	0.157	0.001	-4.2	-4.2
**Heard family planning on television last few months**										
No										
Yes	-0.023	0.201	-0.005	26.9	26.9	-0.036	0.218	-0.008	30.8	30.8
**Heard family planning in newspaper/magazine last few months**										
No										
Yes	0.000	0.299	0.000	-0.7		0.006	0.375	0.002	-8.8	-8.8
**Household characteristics**										
**Wealth Index**										
Poorest										
Poorer	-0.007	-0.347	0.002	-13.8		-0.010	-0.462	0.005	-18.5	
Middle	-0.010	0.119	-0.001	6.8		-0.011	-0.075	0.001	-3.3	
Richer	-0.013	0.544	-0.007	41.7		-0.013	0.338	-0.004	17.6	
Richest	-0.010	0.870	-0.009	53.0	87.8	-0.019	0.776	-0.015	59.8	55.5
**Religion**										
Hindu										
Muslim	0.004	0.054	0.000	-1.2		0.015	0.001	0.000	0.0	
Others	-0.002	0.141	0.000	1.4	0.2	-0.001	0.248	0.000	0.8	0.7
**Caste**										
Scheduled Caste										
Scheduled Tribe	0.003	-0.364	-0.001	5.3		-0.001	-0.412	0.000	-0.8	
Other Backward Class	0.016	0.058	0.001	-5.5		0.006	0.014	0.000	-0.3	
Others	-0.007	0.170	-0.001	6.8	6.6	-0.004	0.225	-0.001	3.4	2.2
**Place of residence**										
Urban										
Rural	0.011	-0.152	-0.002	10.0	10.0	-0.006	-0.232	0.001	-5.6	-5.6
**Regions**										
North										
Central	0.010	-0.091	-0.001	5.5		0.032	-0.161	-0.005	20.2	
East	-0.006	-0.319	0.002	-10.3		0.027	-0.338	-0.009	35.6	
North East	-0.002	-0.228	0.000	-2.5		0.007	-0.226	-0.002	6.4	
West	0.003	0.211	0.001	-3.2		-0.003	0.187	-0.001	2.5	
South	0.002	0.279	0.001	-3.8	-14.2	-0.005	0.209	-0.001	3.8	68.4
				100.0					100.0	
**Calculated CCI**			-0.017					-0.025		
**Actual CCI**			-0.022					-0.058		
**Residual**			-0.005					-0.033		

Generalized CCI (Concentration Index)

## Discussion

The study examined socioeconomic differences in the use of modern contraceptive methods among young and non-young adults in India using NFHS 4 data. A significant contribution of this study is to reveal that the use of modern contraceptives was more concentrated among young women from the poor socioeconomic group in the Indian context. Prevailing prior studies from low and middle-income countries showed the prevalence of modern contraception among adolescent and young women was lower than the prevalence among non-young women [31–33]. In this milieu, our study provides strong evidence of socioeconomic inequality among non-young women compared to young women in non-use of modern contraceptives. This study found existing differences in the non-usage of modern contraceptive methods among the young category and non-young category. In line with earlier research, our study reported that the usage of modern contraception was significantly associated with age and [19] it decreases with age [31, 34, 35]. This higher uptake among younger women has been attributed to effective communication on family planning issues [36]. On the contrary, a study using NFHS data reveals contraception use among married adolescent females has been continuously low in comparison to higher age groups [26]. Women from the non-young category had significantly lower odds of modern contraceptive use than women from the young category. Similar to these findings a study from NFHS data shows that the age group 20–24 years has the highest rate of contraceptive use before first pregnancy, which decreases as one gets older [37]. Earlier researches have depicted similar findings [33, 38]. Apart from age, this study observed that women's educational level influences their usage of modern contraceptives. Higher educational levels and using modern contraceptives are associated among young adults [35, 36]. The non-young women had a higher percentage contribution of educational status (56.8%) in socioeconomic inequality in modern contraception use than young women (-6.4 percent). This same evidence aligns with multiple studies where women's education level was found to be a substantial predictor multiple studies (38-40). A cross-country study including India, Bangladesh, Nepal, and Pakistan on contraceptive use and inherent socioeconomic inequality showed illiteracy, poor economic status, and rural contributed negatively to inequalities in contraceptive use [39]. Likewise, another study including 11 low- and middle-income countries shows inequalities in the prevalence of contraceptive use were higher among poorer, older, and non-educated women [40]. In addition, previous researches also revealed that modern contraception use is linked to education [41], exposure to mass media [20], knowledge about family planning [7], household wealth status [42], surviving son, religion, and ethnicity [43].

This study further reveals that modern contraceptive use is concentrated among women from poor socioeconomic strata both in young and non-young categories. The non-use was more common among women in the highest wealth quintile, the probable reason might be the fear of side effect or health concern [44, 45] among wealthy women[46]. The estimates from this study confirm the concentration quintile of modern contraceptive use had higher inequality among non-young women compared to young women. The reason may be, in concurrence with Sedgh et al. [47], that non-young women may have infrequent sex and are less likely to become pregnant as a cause of non-use. Similar to these findings, other possibilities are, work leading to geographic relocation [47], which can lead to couples living apart may be the reason for non-use among non-young category women. Additionally, some studies found that participants cited "method-related" reasons for not using contraceptives reflecting unhappiness with current contraceptive techniques [46]. Other factors that could explain why women in the highest wealth quintile had a greater mean prevalence of non-use are that non-young women refusing to use contraception may be because of their spouse's choice, other members of their families or communities' issues, or even their religious beliefs [44, 48]. On the contrary, some studies showed richer women were more likely to use modern contraceptives than poorer women. This could be owing to their social level, which includes access to modern health care and education, influencing their wealth [35, 49, 50]. The present study represents the decomposition estimates of about 87.8 and 55.5% of the socioeconomic inequality was explained by the wealth quintile for modern contraceptive use in young and non-young women. However, a study shows women in the poorest wealth quintile had low demand for modern contraceptives and it varied greatly across states of India [51]. Further, the wealth index, site of residence, husband's educational level, women's educational level, and mass media exposure were the key drivers of pro-poor socioeconomic inequalities, according to decomposition analysis data from another study [52].

When we look at the study participants half of the women from the reproductive age group have heard about family planning on television, around thirty percent from newspaper/magazines, and less than twenty percent from the radio. Alike in the Philippines and Myanmar, a study found a robust link between media exposure and family planning use among married and cohabiting women [53]. Our finding is consistent with a study conducted by Rana et al.[54]. Moreover, prior studies suggest that media exposure significantly contributed to the current use of modern contraceptives [20, 55]. Studies from NFHS data suggest that exposure to radio, television, and movies have a significant favourable impact on current contraceptive use and future contraception intentions [20]. Findings revealed media exposure was a significant driver of socioeconomic inequality in both young and non-young women and suggest that mass media campaigns can help promote the use of modern contraceptives [56].

Furthermore, in this study, the region explained roughly -14.2 percent of the observed difference in modern contraceptive use in young and 68.4 percent in non-young women. Similarly, according to a study, specific demographic areas reflecting undereducated, poor, with few or no children, and without their partner's support, and newlywed women noted inequality in the use of modern contraception. For example, as commonly noticed there is a provider restriction in the supply of contraceptives for newlywed women in the state of Uttar Pradesh [57]. Considering that, the challenge of reducing socioeconomic inequality among non-young women compared to young women in non-use of modern contraceptives is much higher, and educational programs should be created with an equitable perspective in order to target these groups. Therefore, findings from the study have demonstrated substantial evidence on the factors affecting the non-use of modern contraceptives like education, exposure to mass media, knowledge about family planning, household wealth status, religion, and ethnicity.

### Limitations

There were some limitations to this study. Given the country's broad social, cultural, and traditional views and practices, the conclusions generated herein may not be applicable to the entire population. The varied group, migration, and intermarriage within, the findings may not have produced definite information on a single tribe or culture. Women self-reported their usage of modern contraception, and the results could be distorted by interviewer bias or social desirability influencing the estimations. However, the presence of a family member during the interview may influence responses in some situations, particularly among young women and those from the conservative places. Due to data constraints, it was not possible to evaluate additional factors that affect the use of contraceptives, including family dynamics, social norms, and the standard of family planning services. The NFHS survey does not capture the duration of contact or the nature of the conversation, a thorough evaluation of the quality of family planning conversations with healthcare practitioners could not be conducted in this study role. Oftentimes, the family planning programs focused on population control aspect in India [58]. For this matter, accessibility to health centers plays a pivotal role and limited access leads to non-use or discontinuation of contraceptive methods [59]. However, due to huge number of missing cases in the concerned variable in the dataset, the role of accessibility of health centers could not be considered in this study. Lastly, cross-sectional survey data can only reveal an association between the outcomes and explanatory variables, not necessarily a causative relationship which needs to be investigated in future research with advanced methods. Future studies based on the latest data of NFHS-5 need to be conducted that focus on more number of factors associated with socioeconomic inequalities in non-use of modern contraceptives among young and non-young married women in India.

## Conclusion

The current findings provide evidence to promote and enhance the use of modern contraceptives by reducing socioeconomic inequality, which is more effective than traditional contraceptives for both young and non–young women. For policy purpose, it is vital to explore a realistic and long-term solution to wealth-based inequalities in reproductive health utilization. In order to dispel misunderstandings about the non-use of modern contraceptives, it is critical to work on awareness as well as to provide a variety of contraceptive choices to fit each woman.

## Data Availability

The study utilizes secondary source of data which is freely available in public domain through dhsprogram.com.
